# Corin is down-regulated and exerts cardioprotective action via activating pro-atrial natriuretic peptide pathway in diabetic cardiomyopathy

**DOI:** 10.1186/s12933-015-0298-9

**Published:** 2015-10-07

**Authors:** Aiming Pang, Yahui Hu, Pengfei Zhou, Guangfeng Long, Xin Tian, Li Men, Yanna Shen, Yunde Liu, Yujie Cui

**Affiliations:** School of Medical Laboratory, Tianjin Medical University, No. 1 Guangdong Road, Hexi District, Tianjin, 300203 China; Hematopoietic Stem Cell Transplantation Center, Institute of Hematology and Blood Diseases Hospital, Peking Union Medical College and Chinese Academy of Medical Sciences, Tianjin, 300020 China

**Keywords:** Corin, Diabetic cardiomyopathy, Atrial natriuretic peptide

## Abstract

**Background:**

Diabetic cardiomyopathy (DCM), a fatal cardiovascular complication of diabetes mellitus, often leads to progressive heart failure, however its pathogenesis remains unclear. Corin, a cardiac serine protease, is responsible for converting pro-atrial natriuretic peptide (pro-ANP) to biologically active atrial natriuretic peptide (ANP). It has been well established that corin deficiency is associated with the progression of hypertension, cardiac hypertrophy and heart failure. However, because the involvement of corin-mediated pro-ANP processing in DCM has not been clarified, this study aims to investigate the role of corin in the pathogenesis of DCM.

**Methods:**

Diabetes mellitus was induced by a single intraperitoneal injection of streptozotocin (STZ 65 mg/kg) to Sprague–Dawley rats (180–220 g). DCM was confirmed by monitoring continuously transthoracic echocardiography every 4 weeks and hemodynamic measurements at 20 weeks. Myocardial disorder and fibrosis were detected by HE staining and Masson’s trichrome staining. The mRNA and protein levels of corin and ANP in rat hearts and cardiomyocytes were determined by quantitative real-time PCR, western blotting and immunohistochemical staining, respectively. H9c2 cardiomyoblasts proliferation was detected by MTT colorimetric assay and viable cell counting with trypan blue. The effect of *Corin*-*siRNA* H9c2 cardiomyoblasts on EA.hy926 cells migration was measured by the wound healing scratch assay.

**Results:**

The corin and ANP expression in mRNA and protein levels was decreased in DCM rat hearts. Corin and ANP levels of neonatal rat cardiomyocytes and H9c2 cardiomyoblasts treated with high glucose were significantly lower than that of normal glucose treated. Precisely, corin and ANP levels decreased in DCM rats at 12, 16, 20 and 33 weeks; neonatal cardiomyocytes and H9c2 cardiomyoblasts treated with high glucose at 36, 48 and 60 h demonstrated significant reduction in corin and ANP levels. *Corin*-*siRNA* H9c2 cardiomyoblasts showed decreased proliferation. Culture supernatants of *Corin*-*siRNA* H9c2 cardiomyoblasts prevented endothelial cell line EA.hy926 migration in the wound healing scratch assay. Furthermore, iso-lectin expression in arteriole and capillary endothelium was down-regulated in DCM rats.

**Conclusions:**

Our results indicate that corin plays an important role in cardioprotection by activating pro-atrial natriuretic peptide pathway in DCM. Corin deficiency leads to endothelial dysfunction and vascular remodeling.

**Electronic supplementary material:**

The online version of this article (doi:10.1186/s12933-015-0298-9) contains supplementary material, which is available to authorized users.

## Background

Diabetes mellitus (DM) is a chronic, complex disease. The prevalence of diabetes mellitus worldwide continues to increase. The total number of people with diabetes mellitus is estimated to rise from 135 million in 1995 to 300 million in 2025 [1]. Diabetic cardiomyopathy (DCM) is a major cardiovascular complication of diabetes, and the leading cause of heart failure and death [2]. DCM is characterized by its effects on cardiac structures and function in the absence of hypertension and coronary artery diseases [3]. However, to date, knowledge in the pathogenesis of DCM is still limited.

Genetic studies have shown that the human CORIN gene encodes a type II transmembrane serine protease called corin, which is primarily expressed in cardiac tissue. Corin acts as an enzyme in the processing of atrial natriuretic peptide hormone, converting pro-atrial natriuretic peptide (pro-ANP) to biologically active atrial natriuretic peptide (ANP) [4–7]. ANP is a cardiac hormone essential for the regulation of blood pressure by promoting salt excretion, decreasing blood volume, and relaxing vascular smooth muscles [8]. In mice, corin deficiency is associated with hypertension and cardiac hypertrophy [9]; reduced sodium excretion and salt-sensitive hypertension are seen in corin knockout mice due to impaired natriuretic peptide processing [10]. Clinically, patients with heart failure often have decreased plasma corin level [11]. From these studies, we can see that corin, undoubtedly, takes on an important role in the development of cardiovascular diseases.

Endothelial dysfunction and vascular remodeling are associated with both diabetes mellitus and diabetic cardiomyopathy [12, 13]. Our previous study indicates that corin could promote uterine spiral artery remodeling [14]. While, ANP and its downstream molecules could induce endothelial cell proliferation, migration and regeneration after vascular injury [15, 16]. Therefore we speculate that corin may be involved in the pathogenesis of diabetic cardiomyopathy through activation of pro-ANP.

In this study, we used streptozotocin (STZ)-induced diabetes animal model, high glucose-induced neonatal rat cardiomyocytes and H9c2 cardiomyoblasts to detect corin and ANP expression in vivo and in vitro. At the same time, we investigated the effect of *Corin*-*siRNA* H9c2 cardiomyoblasts culture supernatants on EA.hy926 cells migration by wound healing scratch assay. Our results indicated that corin exerted cardioprotective action via pro-ANP activating pathway in DCM, meanwhile, corin deficiency was associated with endothelial dysfunction and vascular remodeling.

## Methods

### Induction of the diabetes model

Forty-five male Sprague–Dawley rats (180–220 g) were purchased from the experimental animal center of Academy of Military Medical Sciences (Beijing, China). The animals were housed at 22 ± 2 °C with 12 h light–dark cycles. All care and experimental procedures of animals were in accordance with the guidelines for the Care and Use of Laboratory Animals published by the National Institute of Health and approved by the Animal Care & Welfare Committee of Tianjin Medical University. The rats were randomly divided into two groups: control group and diabetes group. Diabetes group was induced by a single intraperitoneal injection of STZ (Sigma; 65 mg/kg dissolved in 0.1 mol/L citrate buffer, pH 4.5). The control group received the same dose of citrate buffer alone. The two groups received normal chow. Blood glucose levels were measured on day 3 and 7 after STZ or citrate buffer administration by a hand-held glucometer (UltraEasy, Johnson, USA). Rats with random blood glucose (RBG) >16.7 mM in two consecutive examinations were considered as diabetic model. We monitored body weight, blood glucose, and urine glucose every week. The two groups were sacrificed under deep anesthesia (a single intraperitoneal injection of 3 % sodium pentobarbital at the dose of 50 mg/kg body weight) by exsanguinations.

### Echocardiography and hemodynamic measurements

Transthoracic echocardiography was performed by the vivid 3 pro imaging system (GE, USA) in both groups at 4, 8, 12, 16, 20 weeks. Images were obtained from two-dimensional, M-mode, pulsed-wave Doppler imaging. All measurements were the average of six consecutive cardiac cycles and performed by the same operator. Briefly, male SD rats were lightly anaesthetized with 3 % inhaled isoflurane and set in a supine position. The hemithorax of each rat was carefully shaved. Diastolic interventricular septal wall thickness (IVSd), left ventricular posterior wall thickness in diastole (LVPWd), left ventricular internal dimension in diastole (LVIDd), left ventricular internal dimension in systole (LVIDs), fractional shortening (FS %) and left ventricular ejection fraction (EF %) were measured. Mean arterial blood pressure (MABP), maximal rate of rise in LV pressure (+dP/dt), and maximal rate of decline in LV pressure (−dP/dt) of DCM and Ctrl rats at 20 weeks were measured with a manometer-tipped catheter (SPR-320NR, Millar, USA) and recorded by an MP150 system (Biopac Systems, USA).

### Histology and immunohistochemistry

Paraformaldehyde (4 %)-fixed hearts were embedded in paraffin, and cut into 5 μm sections. The extent of myocyte hypertrophy was evaluated by hematoxylin-eosin staining. Interstitial and perivascular fibrosis were evaluated by Masson’s trichrome staining. For immunohistochemical staining, sections were incubated with anti-corin (sc-67179, Santa Cruz Biotechnology Inc.) or anti-ANP (sc-18811, Santa Cruz Biotechnology Inc.) antibody and a secondary antibody conjugated with HRP (horseradish peroxidase). Nuclei were counterstained with haematoxylin. Immuno-reactivity was demonstrated by 3, 3′-diaminobenzidine (DAB, BOSHIDE). Data were collected from at least five rats per study group.

Immunofluorescent studies were carried out in neonatal rat cardiomyocytes and H9c2 cardiomyoblasts on glass coverslips. The antibodies used were as follows: anti-corin antibody (sc-67179, Santa Cruz Biotechnology Inc.), secondary antibody conjugated with FITC (Invitrogen). Capillary and arteriole densities were identified by immunofluorescent staining with iso-lectin B4. Nuclei were stained with DAPI (4′, 6-diamino-2-phenylindole, Invitrogen). Immunostaining images were examined under a confocal microscope (Leica).

### Electron microscopy

Fresh tissues from SD rat left ventricular anterior wall were cut into about 1 mm^3^ blocks, fixed in 2.5 % glutaraldehyde, 0.1 M cacodylate buffer solution for 24 h at 4 °C, and then embedded in an Araldite-Epon812 mixture. Ultrathin sections (50 nm) were prepared and stained with uranyl acetate and lead citrate. The sample slices were examined using a transmission electron microscope (Hitachi, Japan).

### Isolation and culture of neonatal cardiomyocytes

Neonatal rat cardiomyocytes were acquired from the neonate rats (within 48 h). All experimental procedures were approved by the Animal Care & Welfare Committee of Tianjin Medical University. Neonatal rats were decapitated, a midline incision was made through the sternum. The hearts were gently taken out, atriums were cut off. Then ventricles were cutted into 1–1.5 mm^3^ pieces and digested by 7–8 ml 0.125 % trypsin. The enzyme digestion process was performed for 5–7 min each time and repeated about 8 times until all the tissue blocks were digested. Trysinization was terminated with FBS (FBS: trypsin = 1:10). The cell suspensions were centrifuged at 192×*g* for 8 min at 4 °C and resuspended with DMEM containing 10 % FBS. Collected cells were incubated for 1.5 h differential attachment in DMEM with 10 % FBS to reduce non-myocyte contamination, appropriate 5-bromo-2-deoxyuridine was added into the remaining cell suspension, and followed by cultured in 6-well collagen-coated plates. Cardiomyocytes were treated with various glucose levels: 5.5 mM glucose (Ctrl, normal glucose), 5.5 mM glucose plus 19.5 mM mannose (OC, osmotic control) and 25 mM glucose (HG, high glucose). After incubation at 37 °C for 36, 48, 60 h, cells were harvested for either western blot or real-time qPCR.

### Measurement of mRNA by Reverse transcription polymerase chain reaction (RT-PCR) and quantitative real-time PCR

Total RNA was isolated from rat hearts or cultured cardiomyocytes by using TRIzol reagent (Invitrogen). Reverse transcription was performed using FastQuant RT kit (Tiangen, China). The products of RT-PCR for corin and ANP mRNA expression in rat hearts were detected by agarose gel electrophoresis. Real-time qPCR for corin and ANP mRNA expression in rat hearts and cardiomyocytes was performed using the Stratagene Mx3005P (Agilent Technologies) following the manufacturer’s protocol. PCR cycling conditions: 95 °C for 15 min, followed by 40 cycles of 95 °C (10 s), 58 °C (20 s), and 72 °C (30 s). PCR products were confirmed by melting curve analysis using the MxPro QPCR Software. The relative expression of the genes was analyzed with the 2^−ΔΔCt^ method. The specific primer sequences were given below: 5′-TGCCCAAGCGGAAGTGAG-3′ and 5′-GACGGATGGTCCAGGTTGTTT-3′ for corin; 5′-GTACAGTGCGGTGTCCAACA-3′ and 5′-ATCCTGTCAATCCTACCCCC-3′ for ANP; 5′-GGGTGTGAACCACGAGAAAT-3′ and 5′-ACTGTGGTCATGAGCCCTTC-3′ for GAPDH; 5′-GTTGACATCCGTAAAGACC-3′ and 5′-GACTCATCGTACTCCTGCT-3′ for β-actin. The housekeeping gene GAPDH or β-actin was used as an internal control. All of the tests were measured in duplicate.

### Western blot analysis

Rat hearts were homogenized in a lysis buffer containing 20 mmol/L Tris–HCl (pH 8.0), 100 mmol/L NaCl, 1 mmol/L EDTA, 10 % NP-40 (vol/vol) and a protease inhibitor cocktail (1:100 dilution, Sigma). Proteins from neonatal rat cardiomyocytes and H9c2 cardiomyoblasts were prepared by adding RIPA buffer (P0013B, Beyotime Biotechnology, China) supplemented with a protease inhibitor cocktail. Total protein concentrations were measured using a BCA protein assay kit (Pierce, Thermo). Equal amounts of total extracted proteins from rat hearts (100 μg) or cardiomyocytes (40 μg) were separated by SDS-polyacylamide agarose gel electrophoresis (SDS-PAGE) and transferred electrophoretically to polyvinylidene difluoride (PVDF) membranes (Millipore, USA). The blots were subjected to immunoblot analysis with the primary antibodies and then incubated with secondary HRP-conjugated IgG antibodies. The bands were visualized using enhanced chemiluminescence reagent kit (Millipore, USA). Corin antibody was a gift from Dr. Ningzheng Dong (Cyrus Tang Hematology Center, Jiangsu Institute of Hematology). The other antibodies were as follows: ANP antibody (sc-18811, Santa Cruz Biotechnology Inc.), internal control β-actin (sc-47778, Santa Cruz Biotechnology Inc.). The band intensities were quantified by image J Acquisition and Analysis Software.

### Enzyme-linked immunosorbent assay for N-terminal pro-ANP (NT-proANP) in plasma of rats

Plasma NT-proANP level was measured by ELISA kit (ALPCO, USA). The assay was performed according to manufacturer’s instructions. The concentrations of NT-proANP (ng/mL) were detected at 450 nm by spectrophotometry (Bio-Tek Synergy 2, USA) and were calculated by comparing the OD value of the samples to a standard curve. Results were converted to nmol/L and expressed as concentrations relative to the corresponding control group. All of the samples were measured in duplicate.

### Transfection of *Corin*-*siRNA* into H9c2 cardiomyoblasts

Transfections were performed with lipo3000 (invitrogen, USA) according to the manufacturer’s instructions. Specific *Corin*-*siRNA* sequences were synthesized (genepharma, China) and transfected into H9c2 cardiomyoblasts. The sense and antisense strands of *Corin*-*siRNA* sequence 1 were 5′-GCAGUGUAAUGGCUACAAUTT-3′ and 5′-AUUGUAGCCAUUACACUGCTT-3′. The sense and antisense strands of *Corin*-*siRNA* sequence 2 were 5′-GUGGACAUUAUUUGGUUUATT-3′ and 5′-UAAACCAAAUAAUGUCCACTT-3′. H9c2 cardiomyoblasts were transfected for 48 h, the efficiency of corin silencing was assessed by real-time qPCR and western blot analysis. Data were from at least seven dependent experiments.

### Proliferation assay

Cell proliferation was detected by MTT colorimetric assay and viable cell counting with trypan blue. The experiments were repeated three times at least.

MTT colorimetric assay: The H9c2 cardiomyoblasts were cultured in 96-well plates with DMEM containing 10 % FBS. *Corin*-*siRNA* or *negative control*-*siRNA* (*NC*-*siRNA*) was transfected into H9c2 cardiomyoblasts, 48 h later 20 μL of 5 mg/mL MTT solution was added to each well. Cells were cultured for 4 h at 37 °C, then followed by adding 150 μL DMSO. Absorbance value was detected at 490 nm by a microplate reader (Bio-Tek Synergy 2, USA).

Viable cell counting with trypan blue: The H9c2 cardiomyoblasts were cultured in 6-well plates with DMEM containing 10 % FBS. *Corin*-*siRNA* or *NC*-*siRNA* H9c2 cardiomyoblasts were trypsinized, cell suspension and trypan blue were mixed with 9:1 and viable cells were counted by a haemocytometer.

### The wound healing scratch assay

Scratch test was applied to detect the migration and wound healing of EA.hy926 cells. When the cells reached 80 % confluence in 6-well plates, parallel streaks were created by scraping the confluent cell monolayer with a 200 μL sterile pipette tip, then washed three times with PBS. H9c2 cardiomyoblasts were transfected with specific *Corin*-*siRNA* or *NC*-*siRNA* for 48 h, culture supernatants were added into EA.hy926 cells. The cells were cultured at 37 °C in 5 % CO_2_ and pictures of the parallel lines were taken respectively at 0 and 24 h after scratching under the light microscope. The assay was done in duplicate in each group from at least three dependent experiments.

### Statistical analysis

All values are presented as mean ± SD. Western blot densities were analyzed with image J Acquisition and Analysis Software. All histopathological sections were analyzed by an imaging analysis system (NIH). Data between two groups were assessed by unpaired t-tests. Comparisons for three or more groups were done using one-way ANOVA or two-way ANOVA followed by Fisher’s LSD test. Statistical analysis was carried out using the GraghPad Prism software (version 6.01; GraghPad Prism Software, San Diego, CA, USA). P values less than 0.05 were considered to be statistically significant.

## Results

### Characteristics of rats with STZ-induced diabetic cardiomyopathy

**Fig. 1:**
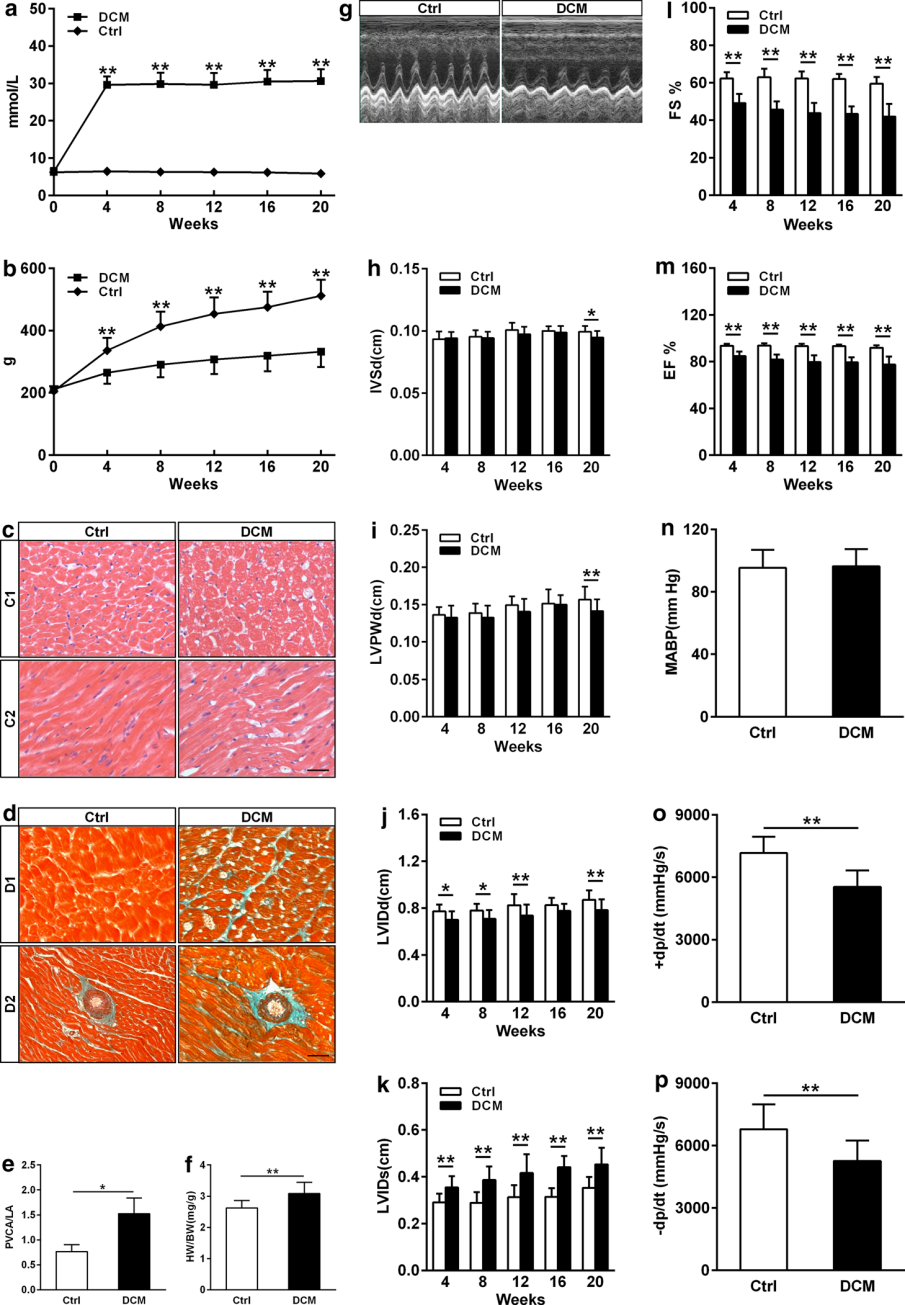
General characteristics of diabetic cardiomyopathy (DCM) and control (Ctrl) rats. **a** Random blood glucose levels (n = 22 to 23 rats for each group). **b** Body weight levels (n = 21 to 23 rats for each group). **c** Cross (*c1*) and longitudinal (*c2*) sections of heart tissues were stained with haematoxylin and eosin (H&E). LV myocyte cross-sectional area was increased in DCM rats in comparison to that in the control group. *Bar* 30 μm. **d** Masson’s trichrome staining showed cardiac interstitial and perivascular fibrosis in DCM rats. *Bar* 30 μm. **e** Perivascular collagen area to lumen area ratio (PVCA/LA) for quantification of Masson’s trichrome staining. Three randomly selected fields from each rat myocardial tissue under ×400 magnification were analyzed using NIH image software (n = 5 rats for each group). **f** Heart weight to body weight ratio (HW/BW, n = 22 rats for each group). **g**–**m** Echocardiographic findings of heart in Ctrl and DCM groups (n = 15 rats for each group). Values are mean ± SD. *P < 0.05 and **P < 0.01 versus Ctrl by two-way ANOVA. **g** Representative images of M-mode echocardiograms. **h** Diastolic interventricular septal wall thickness (IVSd). **i** Left ventricular posterior wall thickness in diastole (LVPWd). **j** Left ventricular internal dimension in diastole (LVIDd). **k** Left ventricular internal dimension in systole (LVIDs). **l** Fractional shortening (FS %). **m** Left ventricular ejection fraction (EF %). **n**–**p** The hemodynamic parameters of heart in DCM and Ctrl groups (n = 11 rats for each group). **n** Mean arterial blood pressure (MABP). **o** Maximal rate of rise in LV pressure (+dP/dt). **p** Maximal rate of decline in LV pressure (−dP/dt). Data are presented as mean ± SD. * P < 0.05 and ** P < 0.01 versus Ctrl

**Fig. 2:**
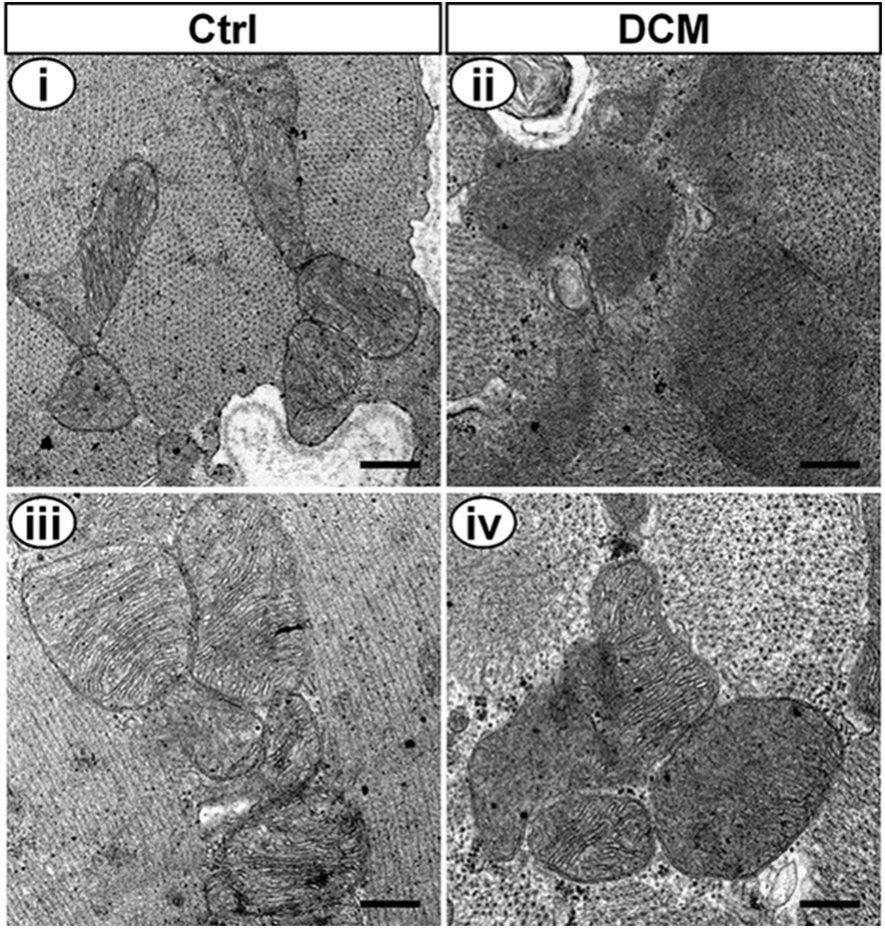
Transmission electron microscopy of Ctrl and DCM rat hearts. *Bar* 400 nm (**i**, **ii**), *bar* 325 nm (**ii**, **iv**). **i**, **iii** The ultrastructure of cardiomyocyte in Ctrl rats showed typical symmetric myofibrils, clear outline of mitochondria, integrated mitochondrial membrane and well-organized cristae. **ii**, **iv** The ultrastructure of cardiomyocyte in DCM rats showed severe mitochondria damage, disordered myofibrils arrangement

DCM rats presented higher blood glucose (22.4–33.3 mmol/L), while that of control group was maintained at normal level (Fig. 1a). Body weights were decreased in DCM rats compared with those in the control group at the same point in time (Fig. 1b). The urine glucose test of DCM rats was consistently positive (Data were not shown). At the same time, haematoxylin eosin staining showed cardiomyocyte hypertrophy and masson’s trichrome staining displayed interstitial and perivascular fibrosis in DCM rats (Fig. 1c–e). Heart weight to body weight ratio was significantly increased in DCM rats in comparison to that in the control group (Fig. 1f). The changes of cardiac structure and cardiac dysfunction were assessed by echocardiography (Fig. 1g–m) and hemodynamic measurements (Fig. 1n–p) in two groups. DCM rats showed impairment of cardiomyocyte including severe mitochondria damage, disordered myofibrils arrangement (Fig. 2) and excess glycogen deposition (Additional file 1: Figure S1) by transmission electron microscopy.

### Corin and ANP levels were decreased in diabetic cardiomyopathy rats

**Fig. 3:**
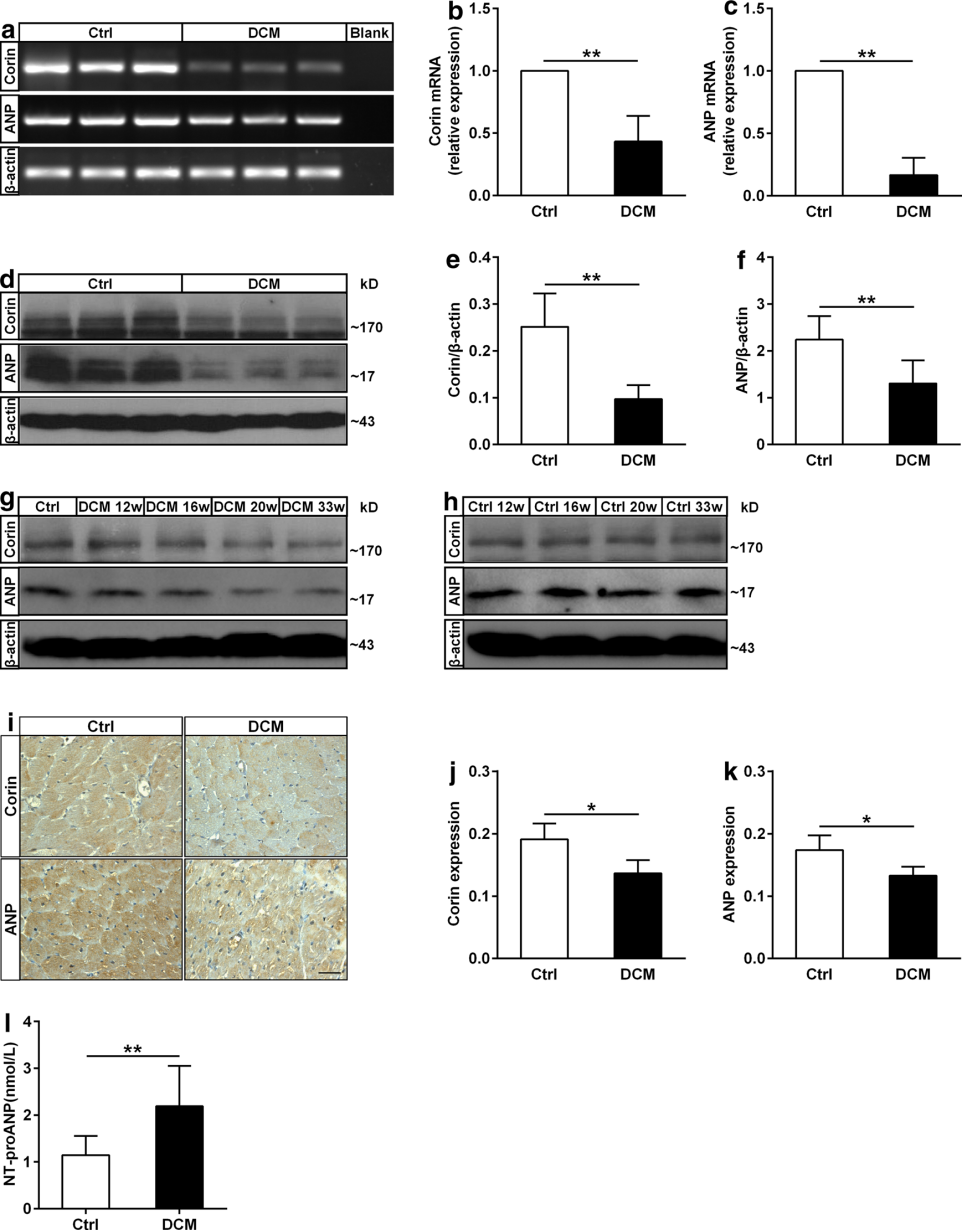
The mRNA and protein levels of corin and ANP in the heart of DCM and Ctrl rats. **a**–**c** The mRNA levels of corin and ANP in hearts of the two groups. **a** RT-PCR analysis of corin and ANP expression. **b**,** c** Quantification for cardiac corin and ANP mRNA levels by real-time qPCR (n = 7 rats for each group in **b**, n = 6 rats for each group in **c**. **d–f** The protein levels of corin and ANP in DCM and Ctrl rats were determined by western blot (n = 7 rats for each group in **e**, n = 8 rats for each group in **f**). **g**, **h** Corin and ANP levels of DCM and Ctrl rats at 12, 16, 20, 33 weeks were measured by western blot. **i**–**k** Corin and ANP protein levels in two groups were evaluated by immunohistochemistry and densitometry analysis. For quantification, at least four randomly selected fields from each rat myocardial tissue under ×400 magnification were analyzed using NIH image software. *Bar* 30 μm (n = 4 rats for each group). **l** The levels of NT-proANP in the plasma of DCM and Ctrl rats were measured by ELISA (n = 15 rats for each group). Data are presented as mean ± SD. *P < 0.05 and **P < 0.01 versus Ctrl

To investigate whether corin and ANP were involved in the progression of DCM, we detected cardiac corin and ANP expression in rats. Reduced corin and ANP expression in mRNA (Fig. 3a–c) and protein (Fig. 3d–f) levels was observed in DCM rats when compared with the control group. Precisely, we found decreased corin and ANP levels in DCM rats at 12, 16, 20, 33 weeks (Fig. 3g) whereas corin and ANP expression in the control rats remained constant (Fig. 3h). Furthermore, immunohistological analysis demonstrated attenuation of corin and ANP expression in DCM rats myocardial pathological sections (Fig. 3i–k). Moreover, we found that plasma NT-proANP level in DCM rats was much higher than that in control group (Fig. 3l). These results indicated that corin was involved in cardioprotection through activation of pro-ANP in DCM.

### The expression of corin and ANP was reduced in high glucose-induced neonatal rat cardiomyocytes and H9c2 cardiomyoblasts

**Fig. 4:**
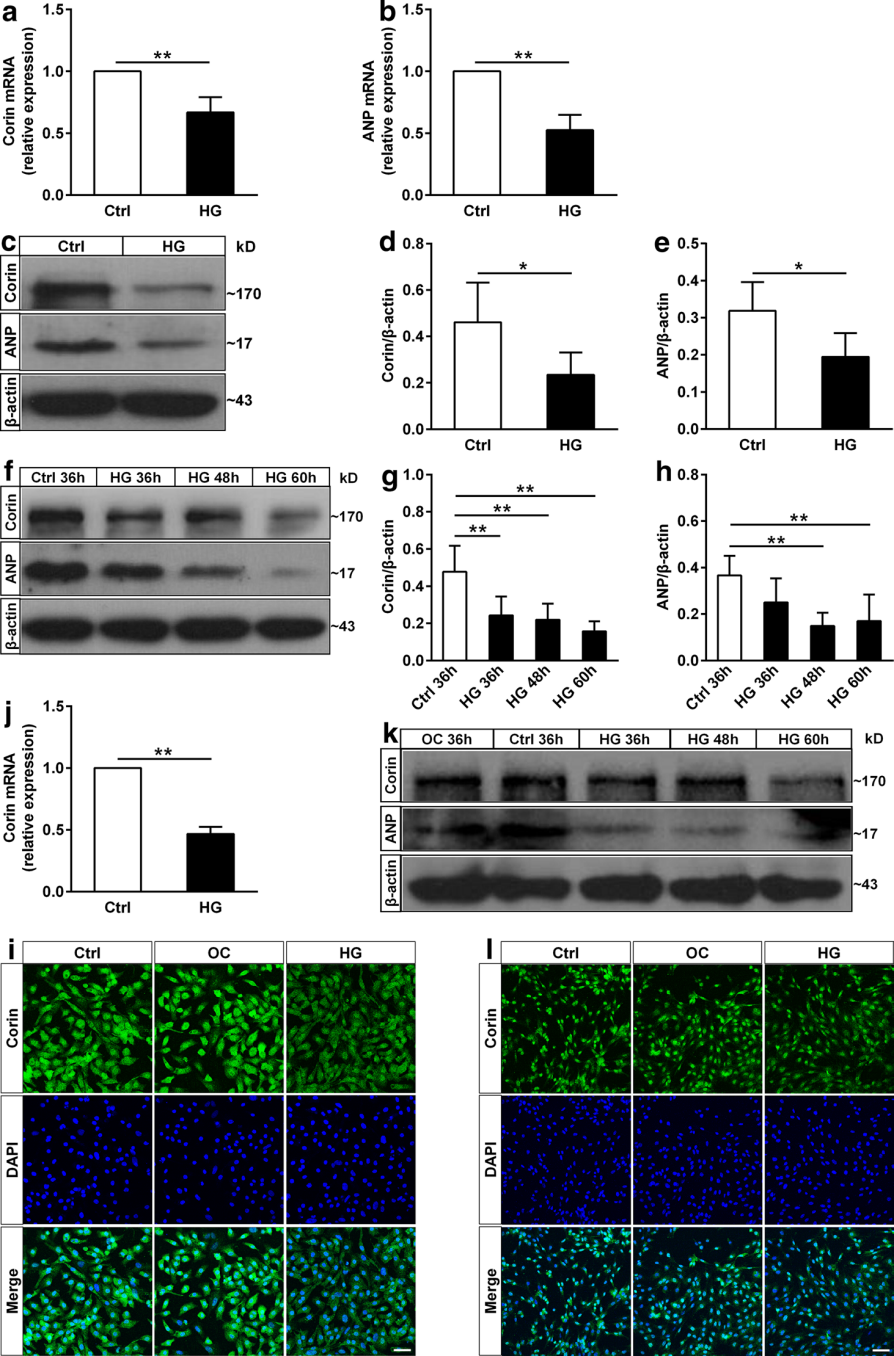
The effect of high ambient glucose on corin and ANP expression in neonatal rat cardiomyocytes and H9c2 cardiomyoblasts.** a**, **b** Quantification for corin (**a**) and ANP (**b**) mRNA levels in neonatal rat cardiomyocytes was measured by real-time qPCR, neonatal rat cardiomyocytes were treated with normal or high glucose for 36 h (n = 6 for each group in **a**, n = 5 for each group in **b**). **c**–**e** The corin and ANP protein levels of neonatal rat cardiomyocytes at 36 h were measured by western blot (n = 6 for each group in **d**, n = 5 for each group in **e**). **f**–**h** Corin and ANP expression in neonatal rat cardiomyocytes at different culture time (n = 4 for each group in **g**, n = 5 for each group in **h**). **i** Immunofluorescence analysis of corin expression in neonatal rat cardiomyocytes at 36 h was performed by confocal fluorescence microscope. *Bar* 40 μm. **j** Corin expression in H9c2 cardiomyoblasts at 36 h was detected by real-time qPCR (n = 6 for each group). **k** Corin and ANP expression in H9c2 cardiomyoblasts at different culture time was determined by western blot. **l** Immunofluorescence analysis of corin in H9c2 cardiomyoblasts at 36 h was performed by confocal fluorescence microscope. *Bar* 80 μm. Ctrl: normal glucose, 5.5 mM glucose; OC: osmotic control, 5.5 mM glucose plus 19.5 mM mannitol; HG: high glucose, 25 mM glucose. Data are presented as mean ± SD. *P < 0.05 and **P < 0.01 versus Ctrl

To further confirm the possible roles of corin and ANP, we next determined corin and ANP expression in vitro. In high glucose-induced neonatal rat cardiomyocytes, corin and ANP expression in mRNA and protein levels was reduced compared with the control group (Fig. 4a–e). Furthermore, corin and ANP protein levels were decreased after neonatal rat cardiomyocytes were treated with high glucose for 36, 48, 60 h (Fig. 4f–h). These results indicated that the levels of corin and ANP in neonatal rat cardiomyocytes were decreased in a time-dependent manner by high glucose treatment. While high osmolarity in neonatal rat cardiomyocytes had no effect on corin and ANP expression (Additional file 1: Figure S2). Immunofluorescence staining also showed that corin expression in high ambient glucose-induced neonatal rat cardiomyocytes was lowered (Fig. 4i). Meanwhile, parallel phenomenon could be also observed in H9c2 cardiomyoblasts (Fig. 4j–l). These data were consistent with the observed corin and ANP expression in DCM rats, suggesting that high glucose level down-regulated expression of corin and ANP in cardiomyocytes.

### Corin played a protective role in cardiomyocytes

**Fig. 5:**
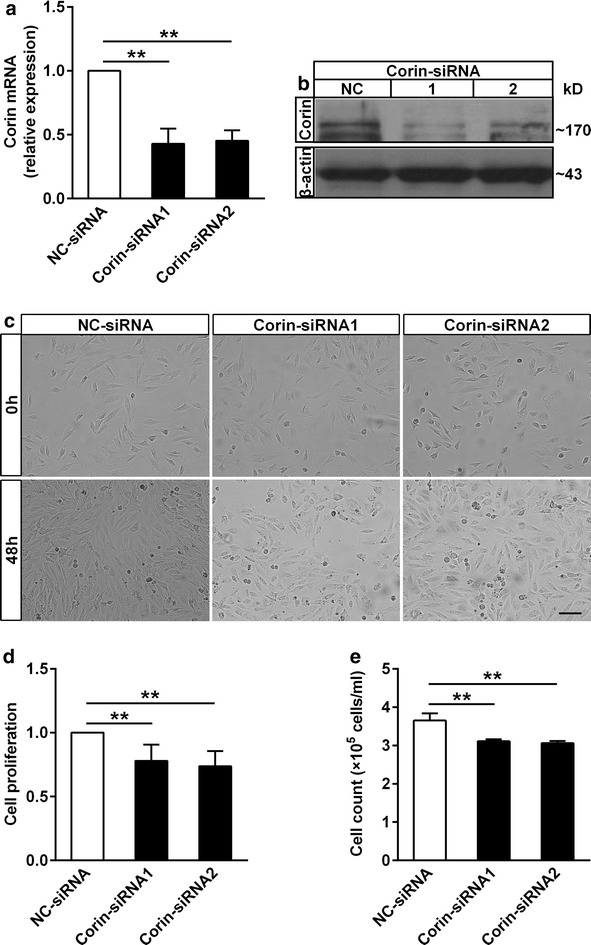
The effect of corin deficiency on H9c2 cardiomyoblasts proliferation. **a**, **b** The silencing efficiency of *Corin*-*siRNA* sequence 1 and sequence 2 in H9c2 cardiomyoblasts was measured by real-time qPCR (n = 6 in each group) and western blot, compared with *negative control*-*siRNA* (*NC*-*siRNA*) group. **c** Morphological alterations of H9c2 cardiomyoblasts proliferation after transfection 0 and 48 h. *Bar* 100 μm. **d** H9c2 cardiomyoblasts were transfected 48 h later, cell proliferation was assayed by MTT (n = 5 in each group). **e** Viable cell count was performed using trypan blue at 48 h. Data represents 3 replicates and 3 repeats. Data are presented as mean ± SD. *P < 0.05 and **P < 0.01 versus *NC*-*siRNA*

To explore the exact role of corin in the development of diabetic cardiomyopathy, we inhibited corin expression by gene silencing of corin. The silencing efficiency of *Corin*-*siRNA* sequence 1 and 2 into H9c2 cardiomyoblasts reached about 55 % by real-time qPCR (Fig. 5a). We further verified that the Corin-siRNA sequences in H9c2 cardiomyoblasts were effective by western blot (Fig. 5b). *Corin*-*siRNA* H9c2 cardiomyoblasts proliferation was inhibited by morphological observation (Fig. 5c). Moreover, we found that the lack of corin significantly decreased cell viability and proliferation in H9c2 cardiomyoblasts when compared with that in the control group using MTT colorimetric assay (Fig. 5d) and viable cell counting with trypan blue (Fig. 5e). These data showed that the cardioprotective effect of corin was attenuated in H9c2 cardiomyoblasts transfected with *Corin*-*siRNA*.

### Defect of corin was associated with endothelial dysfunction and vascular remodeling

**Fig. 6:**
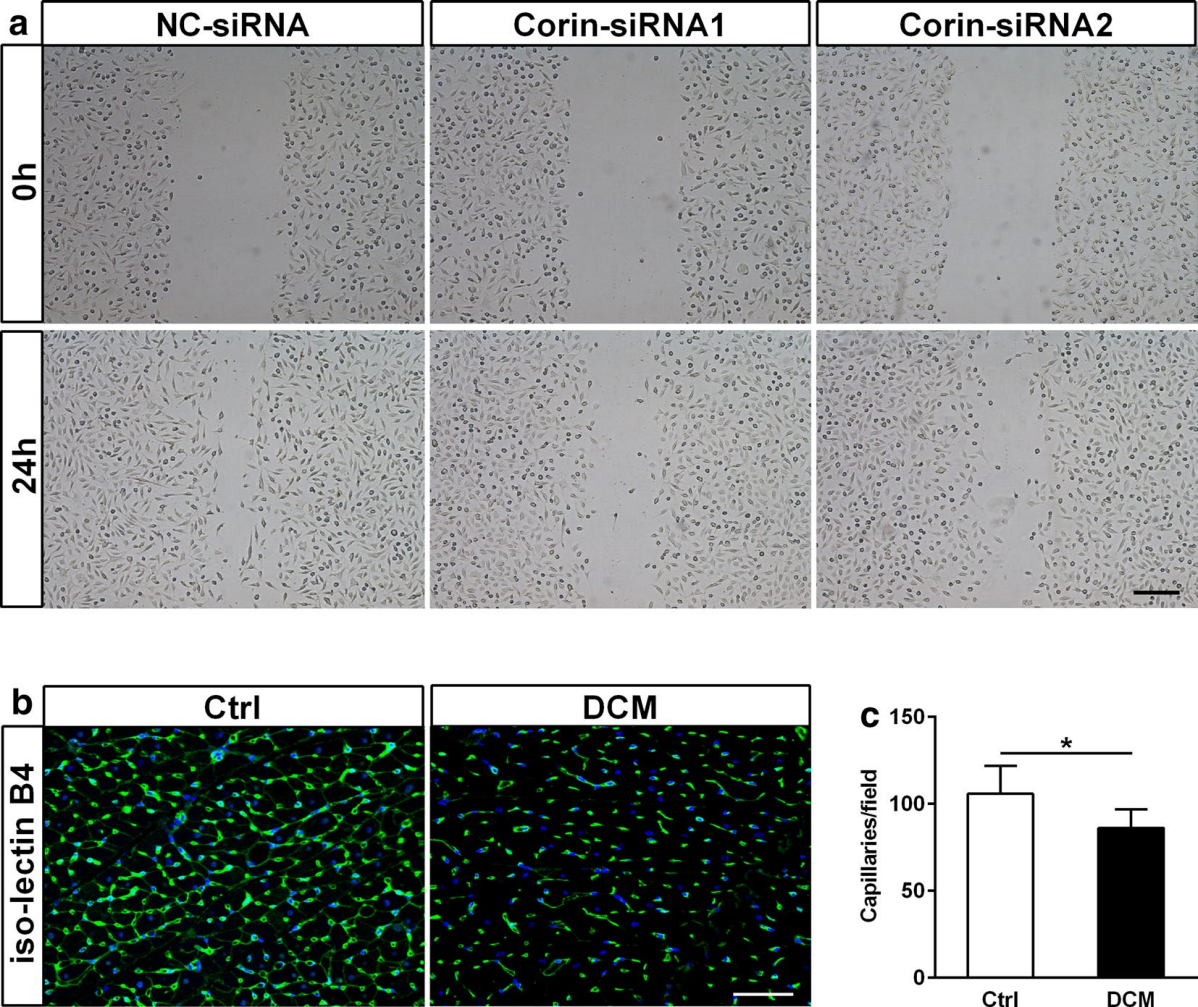
Defect of corin was associated with endothelial dysfunction. **a** The wound healing scratch assay showed the effect of culture supernatants of H9c2 cardiomyoblasts transfected *Corin*-*siRNA* or *NC*-*siRNA* on EA.hy926 cells migration at 0 and 24 h. *Bar* 250 μm. **b**, **c** Reduced capillary and arteriole densities in DCM rat myocardium were detected by confocal fluorescence microscope. Capillary and arteriole densities were stained by iso-lectin B4 (*green*) in rat hearts, nuclei were stained with Dapi (*blue*). *Bar* 50 μm (n = 5 to 6 rats for each group). For quantification, 4 or 5 randomly selected fields from each rat myocardial tissue under ×400 magnification were analyzed. Data are presented as mean ± SD. *P < 0.05 versus Ctrl

To determine the relationship between corin expression and endothelial function, we further detected the effect of *Corin*-*siRNA* H9c2 cardiomyoblasts on EA.hy926 cells migration. The migratory speed of EA.hy926 cells treated with culture supernatants of *Corin*-*siRNA* H9c2 cardiomyoblasts was markedly decreased compared with that in *NC*-*siRNA* group using the wound healing assay (Fig. 6a). These results demonstrated the ability of corin to protect against endothelial dysfunction in vitro. Furthermore, iso-lectin B4 expression in DCM rats was lower than that in the control group (Fig. 6b, c). These findings suggested that the lack of corin impaired endothelial function and ultimately led to vascular remodeling.

## Discussion

In the present study, we demonstrated that cardiac corin expression was decreased in rat DCM models. In addition, downregulation of corin in mRNA and protein levels impaired pro-ANP activating pathway, which led to ANP decline in the DCM hearts. Similar findings were shown in high glucose-induced neonatal rat cardiomyocytes and H9c2 cardiomyoblasts. These results suggested that hyperglycemia inhibited corin-mediated pro-ANP processing in DCM. At the same time, we observed that corin deficiency inhibited H9c2 cardiomyoblasts proliferation and impaired EA.hy926 cells function. This is a novel insight that corin plays a protective role and could maintain endothelial function in DCM.

In our STZ-induced DCM model, plasma glucose levels were increased and hyperglycemia was maintained for a long period of time. Meanwhile, DCM rat hearts exhibited cardiac dysfunction, cardiac hypotrophy, disordered arrangement of muscle fibers, cardiac interstitial and perivascular fibrosis.

### Role of corin in the development of cardiovascular and other diseases

In our study, cardiac corin expression was reduced in both mRNA and protein levels in DCM rats. Consistent with vivo study, corin expression was reduced in high glucose-induced neonatal cardiomyocytes and H9c2 cardiomyoblasts. Thus, we hypothesized that defect of corin may be a contributing factor in DCM. Similar findings of reduced corin level in other cardiovascular diseases have been reported in several studies. Corin deficiency exhibited cardiac hypertrophy and might contribute to hypertensive heart disease in mice [9]. Thomas et al. found that atrial corin mRNA expression was downregulated in rats with heart failure [17]. Plasma corin level was reduced in decompensated heart failure patients [11]. Additionally, corin variant impaired pro-ANP processing, leading to cardiac hypertrophy and hypertension [18]. Low serum corin level predicted adverse cardiovascular prognosis in patients with acute coronary syndrome or after coronary artery bypass grafting surgery [19, 20]. Together, these findings suggested that corin played a cardiac protection effect. In supporting this hypothesis, corin overexpression was shown to improve cardiac function, heart failure, and survival in dilated cardiomyopathy mice [21]. However, Tarazón et al. reported that corin was elevated in heart transplant patients with ischemic cardiomyopathy and LV concentration of corin was inversely related to left ventricular ejection fraction [22]. To this point, we speculate that several possibilities may exist. Firstly, corin expression may differ in different diseases (including acute or chronic). Secondly, corin activity may not increase even with elevated level of corin expression. Another study showed that cardiac corin expression was up-regulated but activity did not increase in late stages of HF patients and the mouse model of HF [23]. Finally, possible corin regulatory mechanisms may exist in cardiac disease. Recently, Chen et al. found that proprotein convertase subtilisin/kexin-6 (PCSK6, also named PACE4) could cleave and activate corin in hypertension [24]. We suppose that regulatory mechanisms for corin may differ in different cardiovascular diseases. At this time, the study of corin is limited, future studies on corin activation will help to better understand corin expression and activity.

Besides cardiovascular diseases, corin is also involved in other diseases. Serum corin levels are reduced in patients with nephrotic syndrome and glomerular disease, osteoporosis and human small cell lung cancer (SCLC) [25–27]. Serum soluble corin level may be a marker or a risk factor for obesity [28]. These findings indicate that corin plays an important role in the development of many diseases.

### Role of ANP in the development of cardiovascular and metabolic diseases

ANP is a cardiac hormone that regulates sodium homeostasis and blood pressure. ANP can be cleaved from pro-ANP by corin. In this study, we found that cardiac ANP expression was decreased. It was consistent with corin downexpression in DCM rat hearts, high glucose induced neonatal cardiomyocytes and H9c2 cardiomyoblasts. Coupled with higher plasma NT-proANP levels in DCM rats, these findings indicated that pro-ANP processing mediated by corin was impaired and cardioprotection of ANP in DCM was attenuated. Similarly, Gutkowska et al. showed that ANP was downregulated in the model of type 2 diabetes [29]. Wang et al. demonstrated that overexpression of ANP slowed HF progression while improved cardiac remodeling and survival in mice with dilated cardiomyophathy [30]; Nakagawa et al. reported that inhibition of aldosterone/MR combined with augmentation of the cardiac ANP/GC-A signaling could prevent the transition of compensated cardiac hypertrophy to HF [31]. ANP exerted protective action against cardiac remodeling and ANP treatment attenuated cardiac inflammation, fibrosis and hypertrophy [32]. These findings suggested that ANP exhibited cardiac protective on many cardiovascular and metabolic diseases. ANP is a useful biomarker in the diagnosis of cardiovascular diseases and a therapeutic agent of cardiovascular diseases [33]. However, a study from Rosa et al. found that ANP expression was increased in spontaneously hypertensive rats with diabetes mellitus [34]. ANP might serve as an important molecule that regulated cardiovascular and metabolic homeostasis [33]. It is possible that ANP expression may differ in different stages of disease.

At the same time, we detected decreased corin and ANP levels in DCM rats at 12, 16, 20, 33 weeks. Consistent with this, corin and ANP levels were significantly reduced after neonatal cardiomyocytes and H9c2 cardiomyoblasts were treated with high glucose for 36, 48, 60 h. Corin and ANP expression was decreased with high glucose treatment in a time-dependent manner, which indicated that the changes of corin and ANP levels were associated with the course of diseases. With these results, we conclude that corin participates in the development of DCM through activation of pro-ANP.

### Relationship between lack of corin and endothelial dysfunction in the development of diabetic cardiomyopathy

Endothelial dysfunction is considered as the pathological basis in cardiovascular diseases and a contributing factor in the progression of diabetic complications [35]. Endothelial dysfunction and vascular remodeling were important in the development of diabetic cardiomyopathy [13, 36]. Here, we found that the lack of corin inhibited endothelial migration in vitro by the scratching test. At the same time, decrease of microvessel density in DCM rat myocardium indicated that the capillary and arteriole endothelium were impaired. Hence, we concluded that the reduced microvessel density was associated with decrease of corin in DCM. Similar findings were reported in our previous study that in corin knockout mice, uterine spiral artery remodeling was impaired, causing pre-eclampsia [14]. In fact, corin activates pro-ANP to ANP. ANP was shown to regulate endothelial cell growth and migration [32], which were important in angiogenic processes [37]. Another study demonstrated that endogenous ANP played a key role in vascular remodeling in ischemic tissue [38]. As shown in our paper, corin and ANP expression were decreased in DCM suggesting that corin deficiency led to endothelial dysfunction and vascular remodeling, which promoted the development of DCM. This is a new molecular mechanism in vascular remodeling of DCM.

In fact, there are several limitations in our study. Firstly, we could not exclude the effects of other factors on DCM due to the lack of corin knockout animal model. Secondly, additional studies are necessary to confirm whether corin improves DCM through other signaling pathway. Finally, corin activator (PCSK6) has been identified recently [25]. Further studies are required to confirm whether PCSK6 is involved in DCM. Nevertheless, our study may provide an important new insight into the pathogenesis of diabetic cardiomyopathy.

## Conclusions

Taken together, we made a significant new finding that cardiac corin and ANP levels were downregulated in DCM. Lack of corin prevented H9c2 cardiomyoblasts proliferation and suppressed endothelial migration in vitro. These data demonstrate that corin plays an important role in cardioprotection by activating pro-ANP pathway in DCM and corin deficiency leads to endothelial dysfunction and vascular remodeling. These findings also indicate that corin can be used as a new therapeutic strategy for DCM.

## Additional file

10.1186/s12933-015-0298-9 Transmission electron microscopy showed excess glycogen accumulation in DCM rats. **Figure S2:** The effect of high osmolarity on Corin and ANP expression levels in neonatal rat cardiomyocytes.
