# Supplementary material for: Corin is down-regulated and exerts cardioprotective action via activating pro-atrial natriuretic peptide pathway in diabetic cardiomyopathy
Source: Cardiovasc Diabetol. 2015 Oct 7;14:134. doi: 10.1186/s12933-015-0298-9 (PMC4597453; doi:10.1186/s12933-015-0298-9)
Supplement: Supplementary file 1 — 10.1186/s12933-015-0298-9 Transmission electron microscopy showed excess glycogen accumulation in DCM rats. Figure S2: The effect of high osmolarity on Corin and ANP expression levels in neonatal rat cardiomyocytes. [file 12933_2015_298_MOESM1_ESM.doc]

**Supplementary figure legends**

**
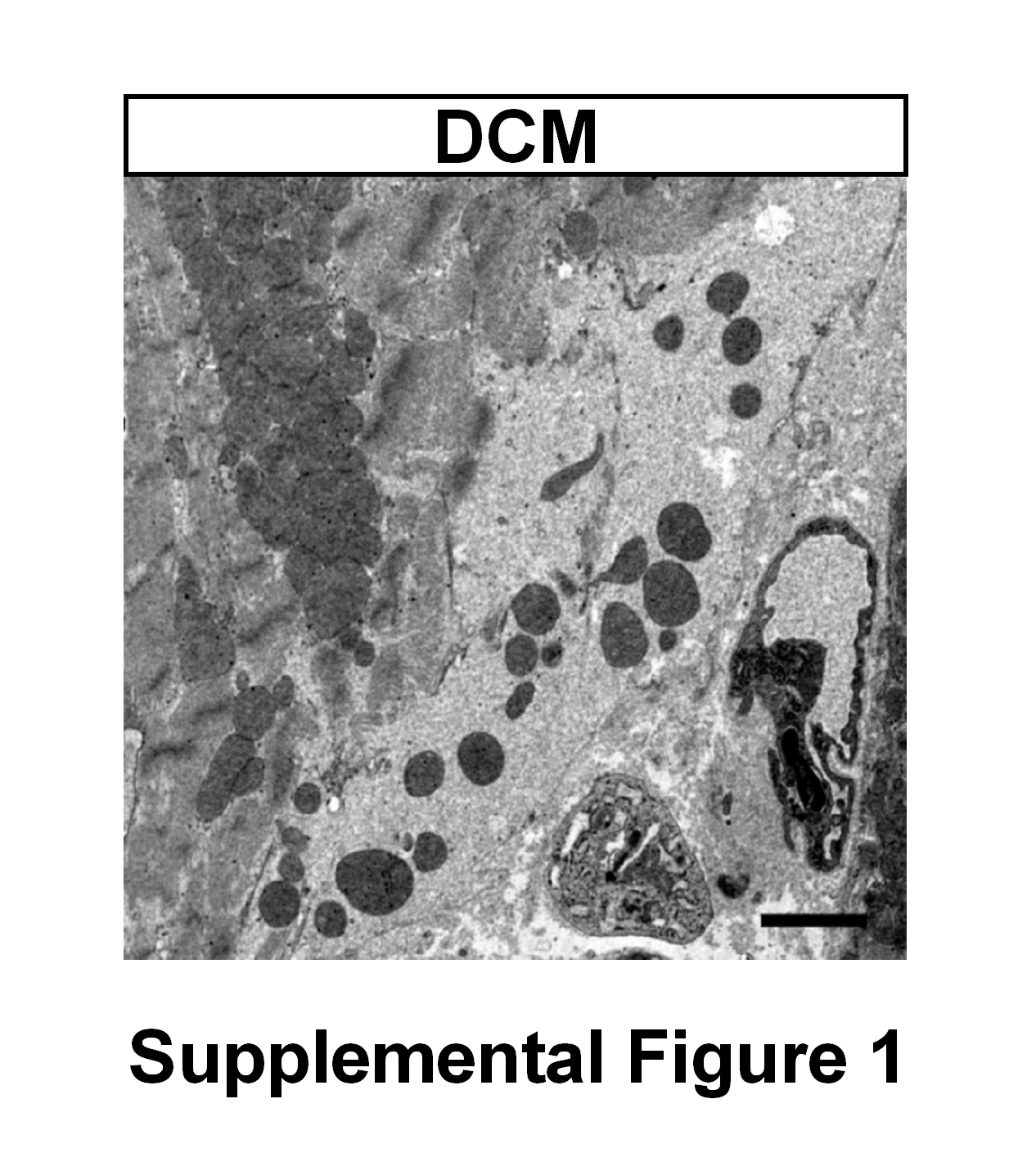
**

**Supplementary figure 1:**

Transmission electron microscopy showed excess glycogen accumulation in DCM rats. Bar= 2.5 μm.


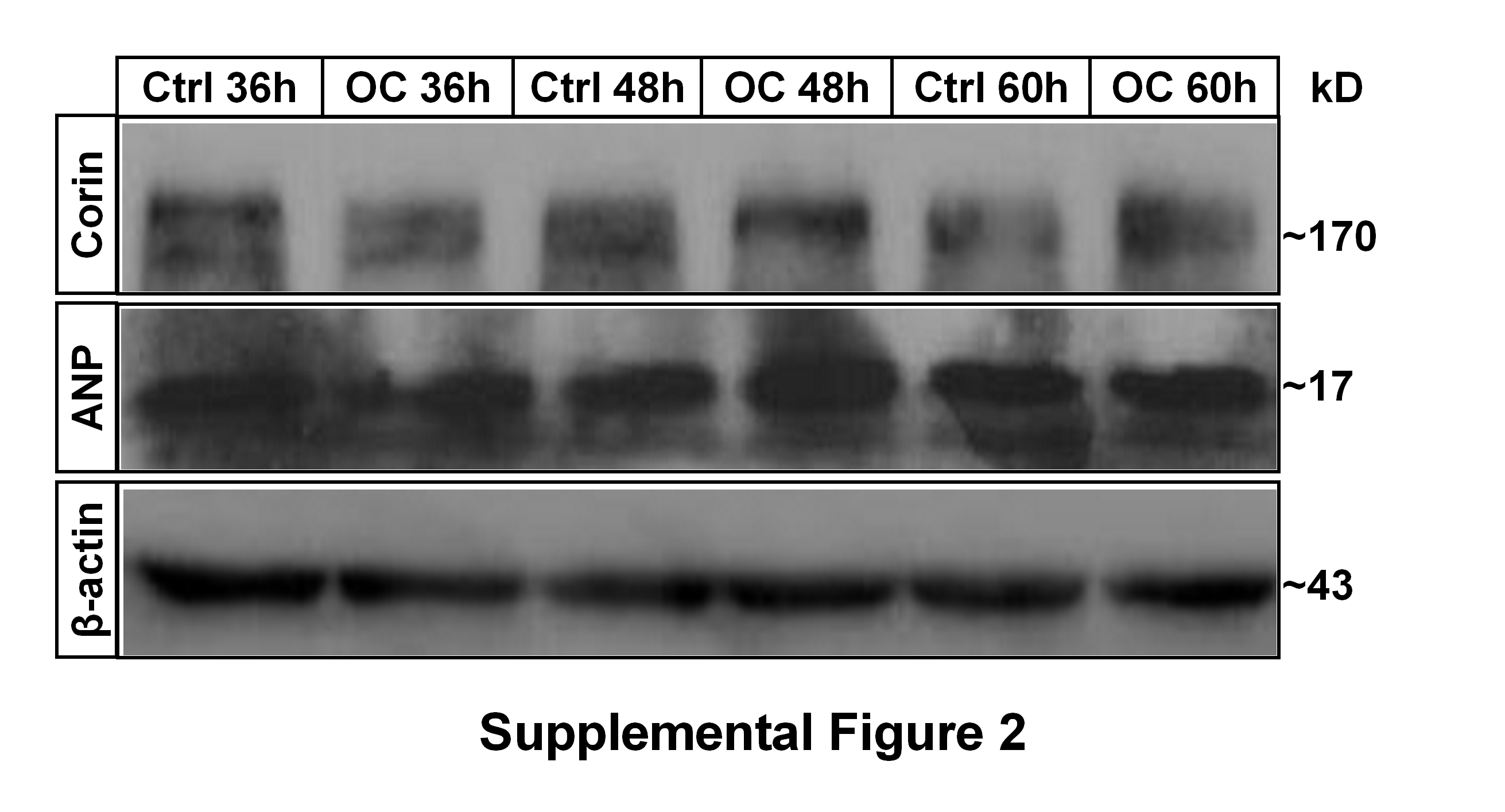


**Supplementary figure 2:**

The effect of high osmolarity on Corin and ANP expression levels in neonatal rat cardiomyocytes. After incubation at 37ºC for 36h, 48h, 60h, neonatal rat cardiomyocytes were harvested for western blot. Ctrl: normal glucose, 5.5 mM glucose; OC: osmotic control, 5.5 mM glucose plus 19.5 mM mannitol.
